# Biomechanical Analysis of Cuboid Osteotomy Lateral Column Lengthening for Stage II B Adult-Acquired Flatfoot Deformity: A Cadaveric Study

**DOI:** 10.1155/2017/4383981

**Published:** 2017-04-10

**Authors:** Haichao Zhou, Haoyang Ren, Chunguang Li, Jiang Xia, Guangrong Yu, Yunfeng Yang

**Affiliations:** ^1^Department of Orthopaedics, Shanghai Tongji Hospital, Tongji University, School of Medicine, Shanghai 200065, China; ^2^Department of Orthopaedics, Yijishan Hospital, Wannan Medical College, Wuhu, Anhui 241000, China

## Abstract

*Purpose*. To investigate the effect of cuboid osteotomy lateral column lengthening (LCL) for the correction of stage II B adult-acquired flatfoot deformity in cadaver.* Methods*. Six cadaver specimens were loaded to 350 N. Flatfoot models were established and each was evaluated radiographically and pedobarographically in the following conditions: (1) intact foot, (2) flatfoot, and (3) cuboid osteotomy LCL (2, 3, 4, and 5 mm).* Results*. Compared with the flatfoot model, the LCLs showed significant correction of talonavicular coverage on anteroposterior radiographs and talus-first metatarsal angle on both anteroposterior and lateral radiographs (*p* < .05). Compared with the intact foot, the above angles of the LCLs showed no significant difference except the 2 mm LCL. In terms of forefoot pressure, medial pressure of the 2 mm LCL (*p* = .044) and lateral pressure of the 3, 4, and 5 mm LCLs showed statistical differences (*p* < .05), but lateral pressure of the 3 mm LCL was not more than the intact foot as compared to the 4 and 5 mm LCLs, which was less than medial pressure.* Conclusion*. Cuboid osteotomy LCL procedure avoids damage to subtalar joint and has a good effect on correction of stage II B adult-acquired flatfoot deformity with a 3 mm lengthening in cadavers.

## 1. Introduction

Adult-acquired flatfoot deformity is mainly caused by posterior tibial tendon dysfunction, which is classified into four stages according to type of deformity by Johnson and Bluman et al. [1, 2]. Nowadays, the clinical treatment usually involves tendon transfer and osteotomies. For stage II B adult-acquired flatfoot deformity, the main pathological changes occur at the transverse tarsal joint with a characteristic of forefoot abduction and hindfoot valgus deformity. So far, the specific treatment still remains controversial. To compensate for the external rotation of the talus and the lateral column [3], simple soft-tissue reconstruction and medializing calcaneal osteotomy are not enough to achieve our expected effect. Thus, an additional surgery of lateral column lengthening (LCL) is needed [4].

The LCL was first described by Evans to correct calcaneal valgus deformity in children [5]. It has been applied as one of the important procedures to correct the flatfoot deformity. The Evans osteotomy was performed in parallel with the calcaneocuboid joint at a distance of approximately 1.5 cm with a wedge-shaped graft lengthening the lateral column; however, the LCL procedure involves the risk of overcorrection, which can also lead to calcaneocuboid osteoarthritis, lateral column foot pain, and fifth metatarsal stress fractures [6–9]. Previous biomechanical experiments have found that internal rotation of the forefoot induced greater pressure on the lateral plantar and calcaneocuboid joint after the LCL procedure [10–12]. Many anatomic studies have demonstrated that the majority of calcanei have a conjoined anterior and middle talocalcaneal articular facet [13, 14]. As a result, the facets are at high risk of articular surface damage during the Evans osteotomy, which means a possibility of complications such as common step-off deformity and pain. To make up for the surgical defects and achieve better outcomes, many surgeons have attempted to change the orientation, distance from the calcaneocuboid joint, and size of the graft based on the Evans osteotomy, but the best surgical option remains controversial [13, 15, 16].

The lateral column of the foot is comprised of the anterior facet of the calcaneus as well as its articulation with the cuboid and the fourth and fifth tarsometatarsal joints. The Evans LCL procedure is performed on the proximal border of the calcaneocuboid joint. To our knowledge, there are no biomechanical reports on the cuboid osteotomy lengthening of the lateral column for flatfoot deformity. Therefore, we performed this study to determine whether this procedure can correct stage II B adult-acquired flatfoot deformity effectively. We use intact cadaveric specimens to establish a flatfoot model and compare the plantar pressures and angulation data in the normal foot, flatfoot, and corrected foot with LCLs.

## 2. Materials and Methods

This study's protocol and amendments were approved by local institutional review boards, and informed consent had been given. The specimens were provided by Tongji University, School of Medicine. Six fresh-frozen intact cadaveric adult feet with no fractures or deformities were used as specimens. There were three male and three female donors. Two were left feet, and four were right. The mean age of the specimen donors was 49.5 (range, 37–61) years. Each specimen was amputated 20 cm from the ankle joint and stored at −20°C. Specimens were thawed at room temperature for 24 hours prior to the test. The skin, subcutaneous tissues, neurovascular bundle, and extensor tendons were removed without injury to the bony structures to allow good exposure of the joints for the osteotomy, protecting the interosseous membrane and joint capsules. The Achilles tendon, peroneus longus (PL), peroneus brevis (PB), flexor digitorum longus (FDL), flexor hallucis longus (FHL), and posterior tibial tendon (PTT) were left for tensile load to create the flatfoot model. The tendons were then grasped with a nonpenetrating Roman-sandal stitch to ensure a strong grip. The proximal tibia and fibula were potted in polymethylmethacrylate for good fixation, using a plumb to keep the axis of the specimen vertical.

This study was designed to simulate double-legged standing stance with the tibia at 90° to the floor. The specimens were loaded on a loading frame (DDL 20, Changchun Academy of Machinery Science & Technology Co. Ltd). The tensile load was applied to each tendon, PTT, 40 N; FDL, 22 N; FHL, 22 N; PBand PL combined, 35 N; and Achilles, 200 N, which was associated with the peak contractile tension of the triceps surae strength percentage, work percentage, and cross-sectional area [11, 17]. Half the body weight of 350 N compressive load was applied on the tibia and fibula. The specimens were preloaded for ten cycles by axially loading to 400 N in order to allow for muscle tensioning and mechanical equilibrium. The specimens were maintained at an axial load of 350 N, and the plantar pressure was recorded by the F-Scan measurement system (Tekscan, Inc.) at a rate of 50 Hz. Each specimen was then attached to a simple load frame with an axial force of 350 N. The frame mainly consisted of two acrylic plates, which facilitated the X-ray fluoroscopy. The talonavicular coverage angle and the talus-first metatarsal angle were measured by the anteroposterior radiographs. The pitch angle, lateral talocalcaneal angle, and talus-first metatarsal angle were measured by the lateral radiographs.

With each specimen, texts conditions were conducted in six conditions: the intact foot, the created flatfoot, and sequential LCL (2, 3, 4, and 5 mm) with cuboid osteotomy. Both plantar pressures and angulation data were recorded in each condition.

**Figure 1:**
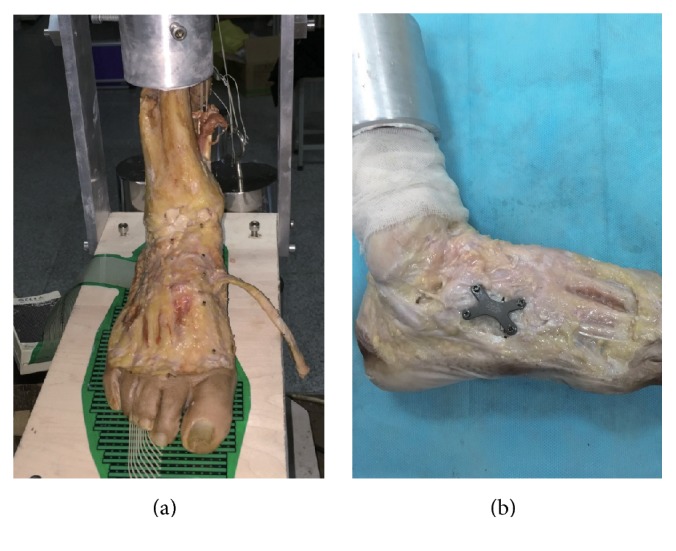
(a) The flatfoot was created and plantar pressure was measured at an axial load of 350 N; (b) cuboid osteotomy lateral column lengthening fixed with a four-hole 2.7 mm plate and four 2.7 mm cortical screws.

We established the flatfoot model by sectioning the talonavicular portion of the superficial deltoid ligament, the long and short plantar ligaments, the spring ligament, and the talocalcaneal interosseous ligament and by releasing the talonavicular capsule as described previously [11, 12]. Loads applied to the tendons remained constant except for PTT, which had no load in order to simulate posterior tibial tendon dysfunction. The foot was axially loaded to 700 N for 200 cycles in order to create stage II B adult-acquired flatfoot deformity (Figure 1(a)). Then, the foot was attached to the simple load frame under X-ray to assess whether the desired deformity had been achieved. If the deformity magnitude did not result, axial loads would be applied every 100 cycles continuously until a talonavicular abduction deformity of 15 to 30 degrees was present under X-ray.

For the LCL procedure, the osteotomy was performed in the middle of the cuboid and parallel with the calcaneocuboid joint. The depth of the osteotomy was 10 mm in order to avoid the influence for the length of the lateral column because of different sizes of cuboid. Attention should be paid to protect the long peroneal tendon, which passed through the tendon groove on the plantar side of cuboid. Metal wedges of four different widths (2, 3, 4, and 5 mm) were customized for this study. These widths were determined from measurements during pilot testing. The other parameters of the wedges were kept the same. The wedge was implanted into the cuboid instead of the bone graft and fixed with a four-hole 2.7 mm plate and 2.7 mm cortical screws (IDEAL), to prevent any migration during loading (Figure 1(b)).

### 2.1. Statistical Analysis

All the radiographic parameters and plantar pressures were assessed in each condition to identify any differences. All data was expressed as mean ± standard deviation, and analysis was conducted with SPSS 20.0 using a one-way repeated measures analysis of variance. LSD was used in pairwise comparison. Significance was set at *p* < .05.

## 3. Results

### 3.1. Angulation Analysis

**Figure 2:**
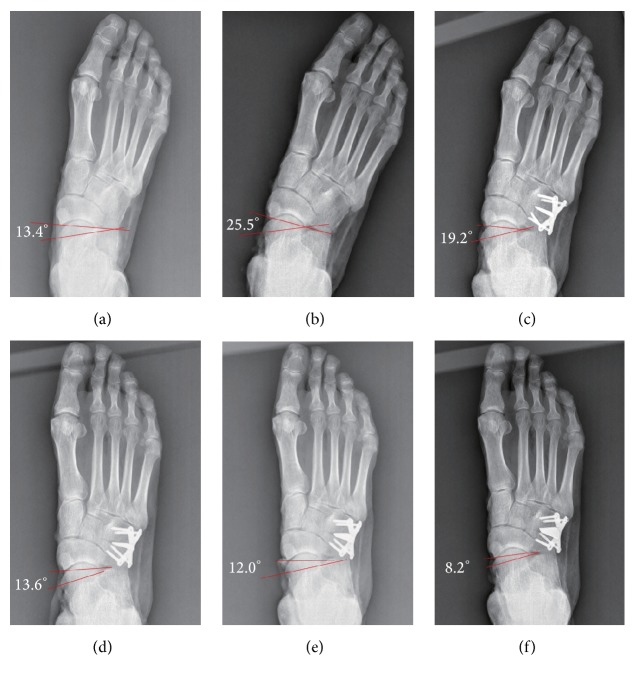
Anteroposterior radiographs of six conditions: (a) intact foot, (b) flatfoot, and (c–f) lengthening of lateral column with cuboid osteotomy (2 mm, 3 mm, 4 mm, and 5 mm).

**Figure 3:**
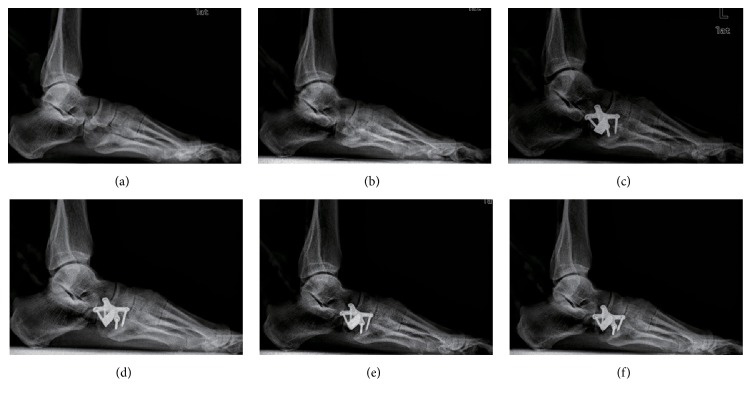
Lateral radiographs of six conditions: (a) intact foot, (b) flatfoot, and (c–f) lengthening of lateral column with cuboid osteotomy (2 mm, 3 mm, 4 mm, and 5 mm).

Compared with the intact foot, anteroposterior talus-first metatarsal angle increased significantly from 8.0° to 19.7° after establishment of the flatfoot model (*p* < .001). On the lateral view, the talus-first metatarsal angle increased from 1.7° to 6.9° (*p* < .001). The talonavicular coverage angle on the anteroposterior radiographs increased from 10.4° to 23.6° as well (*p* < .001). Pitch angle and lateral talocalcaneal angle showed no statistically significant differences with cuboid osteotomy LCL procedure as compared to the intact foot and the flatfoot conditions. Anteroposterior and lateral talus-first metatarsal angle and talonavicular angle decreased as the width of wedges increased, which was significantly different from the flatfoot condition. As the graft increased incrementally, the angles showed no statistically significant difference except 2 mm LCL compared with the intact foot (Table 1, Figures 2, 3, and 5(a)–5(f)).

### 3.2. Plantar Pressure Analysis

**Figure 4:**
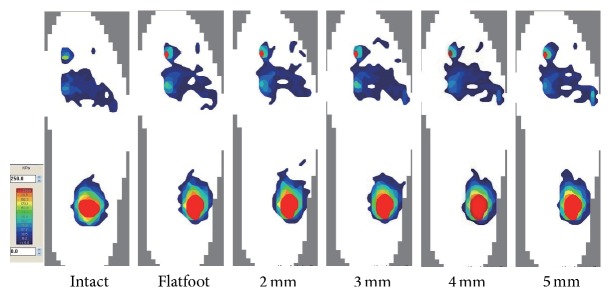
Representative plantar pressure recordings in six different conditions were shown.

**Figure 5:**
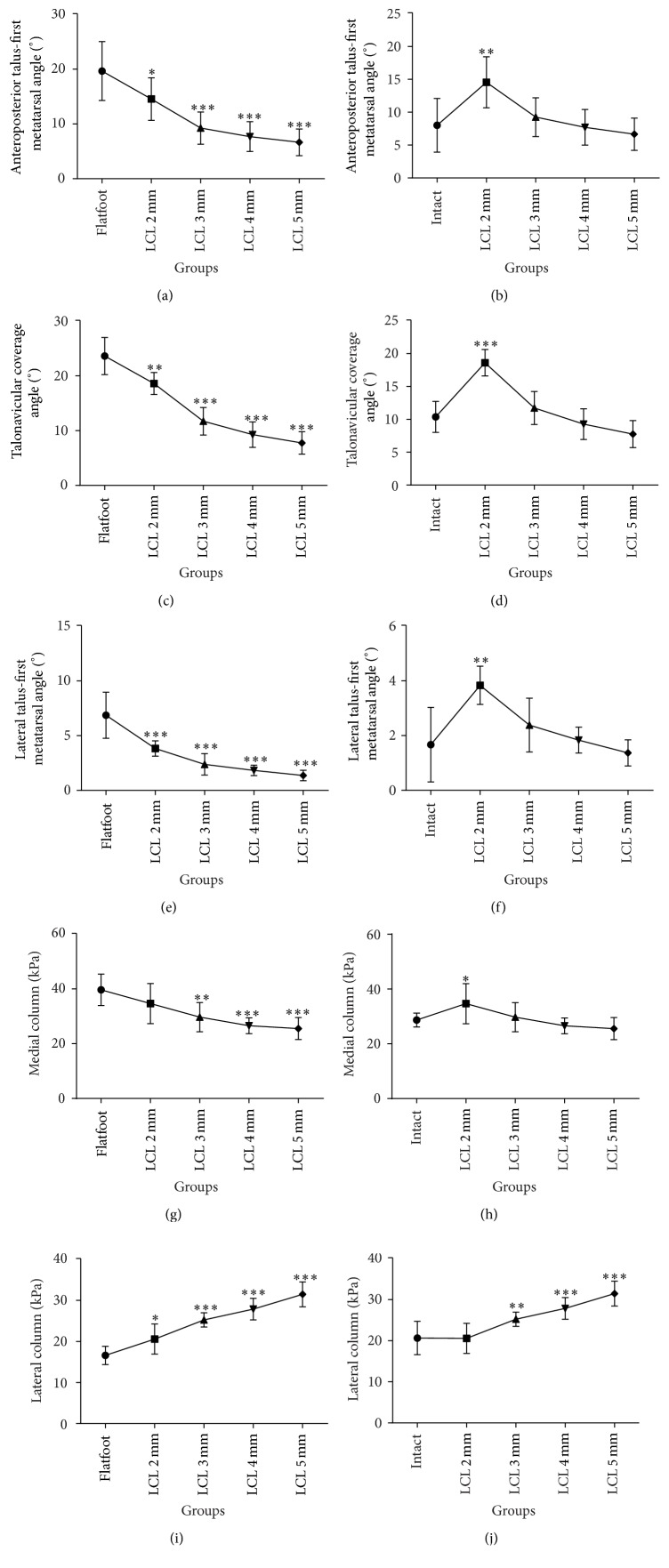
LCL 2, 3, 4, and 5 mm compared with flatfoot (a) and intact foot (b) on anteroposterior talus-first metatarsal angle; LCL 2, 3, 4, and 5 mm compared with flatfoot (c) and intact foot (d) on talonavicular coverage angle; LCL 2, 3, 4, and 5 mm compared with flatfoot (e) and intact foot (f) on lateral talus-first metatarsal angle; LCL 2, 3, 4, and 5 mm compared with flatfoot (g) and intact foot (h) on pressure of medial column; LCL 2, 3, 4, and 5 mm compared with flatfoot (i) and intact foot (j) on pressure of lateral column. Reference: ^*∗*^*p* < .05, ^*∗∗*^*p* < .01, ^*∗∗∗*^*p* < .001.

Only the pressure of the forefoot was analyzed in the present study. We divided the forefoot into medial column and lateral column by the axis of the third metatarsal while performing analysis. After the establishment of the flatfoot model, the average pressure of the lateral column decreased significantly from 20.7 to 16.6 kPa (*p* = .026), and that of the medial column increased from 28.7 to 39.7 kPa (*p* = .001). As the width of wedges increased, the average pressure of the lateral column of the forefoot increased as well, which was even higher than the medial plantar pressure. There was a statistical difference in the pressure of the lateral column compared with that of the flatfoot. No significant difference was observed in the average pressure of the medial column as compared with the flatfoot (*p* = .089), which was statistically different from the intact foot (*p* = .044). As the width of the wedge increased, the average pressure of the medial column gradually decreased and the differences were significant as compared with the flatfoot, which showed no statistical difference from the intact foot. The average lateral plantar pressure showed an excessive increase compared with the intact foot when the width was over 3 mm (Table 2, Figures 4 and 5(g)–5(j)).

## 4. Discussion

The results in this study showed that the flatfoot model was established successfully. Anteroposterior talus-first metatarsal angle and the talonavicular coverage angle increased significantly compared with the intact foot, which indicated an abduction deformity of the forefoot and a valgus deformity of the hindfoot. The increment of the lateral talus-first metatarsal angle showed that our flatfoot model was a mild one. There were no statistical differences of pitch angle or lateral talocalcaneal angle between the intact and the flatfoot conditions, which was similar to the previous cadaveric study [17]. After the cuboid osteotomy LCL procedure, the abduction deformity of the forefoot was corrected effectively because the anteroposterior talus-first metatarsal angle and the talonavicular coverage angle decreased significantly. Meanwhile, our results also showed that both angles would be less than those in the intact foot with the risk of overcorrection if the width of wedges was over 3 mm. Oh et al. [10] found that the talonavicular coverage angle was corrected by approximately 4° with each 2 mm increase in Evans LCL. Chan et al. also reported that about 2-3° or 2% of the talonavicular coverage angle was corrected per millimeter of LCL clinically [18]. However, Benthien et al. [17] found that the talonavicular coverage angle decreased from 46° to 24° with a 10 mm LCL in the flatfoot model. They achieved much greater correction than others possibly because the flatfoot models ranged widely in severity, resulting in differences of the corrective angle. In the present study, the talonavicular coverage angle changed 5° in the 2 mm LCL condition, and the corrective angle would increase to 11.9° if the width of the wedge was increased to 3 mm. The study demonstrated that a large correction of angle could be achieved for the forefoot with a short lengthening of the lateral column by cuboid osteotomy LCL procedure.

In terms of plantar pressure, there were some differences in the midfoot between cadaver specimens and patients with flatfoot deformity because the specimens were unable to compensate for the pressure like the live patients could, but a change of trend still existed. We found that the pressure of the medial column increased significantly, while that of the lateral column decreased after the establishment of the flatfoot model, which was statistically different from the intact foot. As the graft increased incrementally, lateral plantar pressures increased gradually and medial plantar pressures decreased at the same time, similar to the results of prior studies on the Evans osteotomy [10, 19, 20]. In this study, the pressure of the lateral column was approximately equal to that of the intact foot with a 2 mm LCL, while the pressure of the medial column was 34.7 kPa, which showed no statistical difference compared with the flatfoot condition. As the width of the wedge increased to 3 mm, medial plantar pressure decreased significantly and the pressure of the lateral column increased to 25.3 kPa. Moreover, the lateral pressure would be much higher than in the intact foot if the width continued to increase, leading to a high risk of stress fractures.

Evans LCL procedure was recommended for the treatment of stage II B posterior tendon dysfunction because it played a major role in correcting forefoot abduction deformity. In consideration of its possible complications and medial damage, many biomechanical experiments have been conducted. Raines Jr. and Brage [21] performed an anatomic study and found that an osteotomy 15 mm from the calcaneocuboid may injure the FHL, FDL, medial plantar nerve, or tibialis posterior. They suggested a 10 mm interval to be safe. Mosca [22] found that making an osteotomy from proximal-lateral to distal-medial could protect the two facets of the subtalar joint. Although there seems to be some controversy about how to improve the Evans procedure, surgeons have realized the importance of the Evans osteotomy passing between the anterior and middle calcaneal facets [14, 22]. However, in 1904 Laidlaw [23] performed an anatomic study with 750 calcanei and found that 68% had combined anterior and middle facets while only 32% had distinct facets. In 2002 Hyer et al. [13] conducted a surgical anatomy study of 768 calcanei and found that the mean width of facet separation was only 3.85 mm. It is difficult for an Evans osteotomy to avoid damaging the facets. What is more, the talus obscures the view of the depths of the surgical site. An osteotomy is likely to cause step-off deformity, arthrofibrosis, and pain [9]. As alluded to earlier, the author believes that the Evans LCL is not fit for all cases of stage II B adult-acquired flatfoot.

As an important part of the lateral column, cuboid maintains the stability of the lateral longitudinal arch. It is pyramidal in shape, with the base located medially and the apex laterally. It has articular surfaces medially, proximally, and distally. The medial facet of the cuboid articulates with the lateral cuneiform, and the proximal facet of the cuboid articulates with the calcaneus. The distal articular surface has a medial facet that articulates with the base of the fourth and fifth metatarsals [24]. Ligaments around the bone structure are also relatively stable. Based on the anatomical features of the cuboid, a cuboid osteotomy LCL would have a lower risk of articular surface damage than the Evans procedure. Moreover, we have found that the procedure was able to correct an abduction deformity of the forefoot in cadaver according to the radiographic parameters and plantar pressures just as well as an Evans LCL [10, 17]. The results of this study prove the feasibility of the procedure. Tien et al. [25] reported that the capacity of the Evans LCL was occasionally dissipated by subluxation through the retained calcaneocuboid motion segment, so there was need for larger grafts to correct the deformity. This procedure was able to achieve similar treatment effects as an Evans osteotomy but with shorter lengthening: the size of the bone wedge used for the Evans osteotomy ranged from 6 to 10 mm clinically, and 8 mm LCL seemed to be the best because the calcaneocuboid joint pressure was most similar to the intact foot. With the increment of the wedge, the pressure of the forefoot lateral column was overloaded. For the cuboid osteotomy LCL, the sizes of the wedge used in our study were 2, 3, 4, and 5 mm, and 3 mm was the most appropriate according to the radiographic parameters and plantar pressures. Both of the methods had a limited size of the wedge, and they surely had good effects on the correction of the abduction deformity. Differently, the Evans osteotomy might have an influence on the hindfoot valgus deformity, and the cuboid osteotomy was better to correct the forefoot deformity. Meanwhile, the latter prevented an excessive increase of lateral plantar pressure due to the lengthening of the lateral column. Most importantly, though, the advantage of this procedure was that it does not injure the subtalar joint, avoiding the damage to the arm of the sustentaculum tali as well.

We admit that there are several limitations to our study. First, the created flatfoot model differed to some extent from clinical flatfoot deformities. It was difficult to simulate severe adult-acquired stage II B flatfoot deformity completely, so we created mild flatfoot models and analyzed the changing trends of plantar pressures and angles. Second, the results of this study were limited by small sample size. Also, some errors occurred in the tensile load of tendons due to friction between the suture and the load frame. Third, we chose metal wedges to replace graft bones; so no comparisons were made between wedges of different shapes such as triangular or rectangular grafts [12]. There may be some effect on the correction of deformity. In addition, this study focused solely on cuboid osteotomy LCL; the Evans LCL procedure was not done by contrast. Since these two procedures were performed on the same foot, they impacted each other, which may have influenced the authenticity of the results. Previous studies have revealed that calcaneocuboid joint pressure varies with the incremental lengthening of the lateral column. Our study still needs to be improved to analyze the effect of cuboid osteotomy LCL in the calcaneocuboid joint pressure. Finally, other procedures for the correction of flatfoot deformity, such as a medializing calcaneal osteotomy, a soft-tissue balancing procedure, and a tendon transfer, which restores function to the medial column as well as increasing the height of the medial arch, were not added to the LCL. The most appropriate lengthening of cuboid osteotomy was just for the LCL procedure because the medializing calcaneal osteotomy and other soft-tissue procedures would also change the lateral pressure of the forefoot.

In conclusion, the Evans LCL procedure still plays a major role in restoring the abduction of the forefoot in the treatment of stage II B adult-acquired flatfoot deformity. In consideration of potential risks and postoperative complications, we demonstrated a new method of cuboid osteotomy LCL. This procedure was shown to have a good effect on the correction of stage II B adult-acquired flatfoot deformity with a 3 mm lengthening in cadavers.

## Figures and Tables

**Table 1:** Comparison of radiographic parameters.

	Intact	Flatfoot	LCL 2 mm		LCL 3 mm		LCL 4 mm		LCL 5 mm	
Anteroposterior talus-first metatarsal angle (°)	8.0 ± 4.1	19.7 ± 5.4 (*p* < .001)	14.6 ± 3.9	*p* = .023 *p*^*∗*^ = .005	9.3 ± 2.9	*p* < .001 *p*^*∗*^ = .569	7.7 ± 2.7	*p* < .001 *p*^*∗*^ = .859	6.7 ± 2.4	*p* < .001 *p*^*∗*^ = .528

Talonavicular coverage angle (°)	10.4 ± 2.4	23.6 ± 3.4 (*p* < .001)	18.6 ± 2.0	*p* = .001 *p*^*∗*^ < .001	11.7 ± 2.5	*p* < .001 *p*^*∗*^ = .354	9.3 ± 2.3	*p* < .001 *p*^*∗*^ = .449	7.8 ± 2.1	*p* < .001 *p*^*∗*^ = .078

Lateral talus-first metatarsal angle (°)	1.7 ± 1.4	6.9 ± 2.1 (*p* < .001)	3.8 ± 0.7	*p* < .001 *p*^*∗*^ = .003	2.4 ± 1.0	*p* < .001 *p*^*∗*^ = .296	1.8 ± 0.5	*p* < .001 *p*^*∗*^ = .806	1.4 ± 0.5	*p* < .001 *p*^*∗*^ = .659

Talocalcaneal angle (°)	46.4 ± 2.7	45.3 ± 4.4 (*p* = .651)	45.1 ± 5.3	*p* = .933 *p*^*∗*^ = .592	45.8 ± 4.2	*p* = .845 *p*^*∗*^ = .796	45.1 ± 4.1	*p* = .928 *p*^*∗*^ = .587	45.0 ± 3.5	*p* = .895 *p*^*∗*^ = .559

Pitch angle (°)	25.7 ± 2.1	23.5 ± 2.4 (*p* = .058)	24.3 ± 2.0	*p* = .500 *p*^*∗*^ = .207	24.5 ± 1.6	*p* = .304 *p*^*∗*^ = .362	25.0 ± 1.3	*p* = .183 *p*^*∗*^ = .549	25.2 ± 1.2	*p* = .054 *p*^*∗*^ = .976

Flatfoot *p* values compare flatfoot with intact; remaining *p* values compare corrected foot with flatfoot; *p*^*∗*^ values compare corrected foot with intact.

**Table 2:** Comparison of pressure of forefoot.

	Intact	Flatfoot	LCL 2 mm		LCL 3 mm		LCL 4 mm		LCL 5 mm	
Medial column (kPa)	28.7 ± 2.6	39.7 ± 5.8 (*p* = .001)	34.7 ± 7.3	*p* = .089 *p*^*∗*^ = .044	29.7 ± 5.3	*p* = .001 *p*^*∗*^ = .720	26.6 ± 2.9	*p* < .001 *p*^*∗*^ = .464	25.5 ± 4.0	*p* < .001 *p*^*∗*^ = .281

Lateral column (kPa)	20.7 ± 4.1	16.6 ± 2.2 (*p* = .026)	20.6 ± 3.7	*p* = .029 *p*^*∗*^ = .962	25.2 ± 1.7	*p* < .001 *p*^*∗*^ = .013	27.9 ± 2.6	*p* < .001 *p*^*∗*^ < .001	31.5 ± 3.0	*p* < .001 *p*^*∗*^ < .001

Flatfoot *p* values compare flatfoot with intact; remaining *p* values compare corrected foot with flatfoot; *p*^*∗*^ values compare corrected foot with intact.
